# Associations of [^18^F]-APN-1607 Tau PET Binding in the Brain of Alzheimer’s Disease Patients With Cognition and Glucose Metabolism

**DOI:** 10.3389/fnins.2020.00604

**Published:** 2020-06-30

**Authors:** Jiaying Lu, Weiqi Bao, Ming Li, Ling Li, Zhengwei Zhang, Ian Alberts, Matthias Brendel, Paul Cumming, Huimeng Lu, Zhenxu Xiao, Chuantao Zuo, Yihui Guan, Qianhua Zhao, Axel Rominger

**Affiliations:** ^1^PET Center, Huashan Hospital, Fudan University, Shanghai, China; ^2^Department of Nuclear Medicine, University Hospital Bern, Bern, Switzerland; ^3^Department of Nuclear Medicine, University Hospital of Munich, Ludwig Maximilian University of Munich, Munich, Germany; ^4^Faculty of Health, School of Psychology and Counselling, Queensland University of Technology, Brisbane, QLD, Australia; ^5^Department of Neurology, Huashan Hospital, Fudan University, Shanghai, China

**Keywords:** tau, neurodegeneration, metabolism, cognition, Alzheimer’s disease, positron emission tomography

## Abstract

Molecular imaging of tauopathies is complicated by the differing specificities and off-target binding properties of available radioligands for positron emission tomography (PET). [^18^F]-APN-1607 ([^18^F]-PM-PBB3) is a newly developed PET tracer with promising properties for tau imaging. We aimed to characterize the cerebral binding of [^18^F]-APN-1607 in Alzheimer’s disease (AD) patients compared to normal control (NC) subjects. Therefore, we obtained static late frame PET recordings with [^18^F]-APN-1607 and [^18^F]-FDG in patients with a clinical diagnosis of AD group, along with an age-matched NC group ([^18^F]-APN-1607 only). Using statistical parametric mapping (SPM) and volume of interest (VOI) analyses of the reference region normalized standardized uptake value ratio maps, we then tested for group differences and relationships between both PET biomarkers, as well as their associations with clinical general cognition. In the AD group, [^18^F]-APN-1607 binding was elevated in widespread cortical regions (*P* < 0.001 for VOI analysis, familywise error-corrected *P* < 0.01 for SPM analysis). The regional uptake in AD patients correlated negatively with Mini-Mental State Examination score (frontal lobe: *R* = -0.632, *P* = 0.004; temporal lobe: *R* = -0.593, *P* = 0.008; parietal lobe: *R* = -0.552, *P* = 0.014; insula: *R* = -0.650, *P* = 0.003; cingulum: *R* = -0.665, *P* = 0.002) except occipital lobe (*R* = -0.417, *P* = 0.076). The hypometabolism to [^18^F]-FDG PET in AD patients also showed negative correlations with regional [^18^F]-APN-1607 binding in some signature areas of AD (temporal lobe: *R* = -0.530, *P* = 0.020; parietal lobe: *R* = -0.637, *P* = 0.003; occipital lobe: *R* = -0.567, *P* = 0.011). In conclusion, our results suggested that [^18^F]-APN-1607 PET sensitively detected tau deposition in AD and that individual tauopathy correlated with impaired cerebral glucose metabolism and cognitive function.

## Introduction

The hyperphosphorylated, aggregated tau that comprises intracellular filamentous inclusions is implicated in a number of neurodegenerative pathologies (Spillantini and Goedert, 2013). In healthy adults, equal amounts of tau protein isoforms with three microtubule-binding domains (3R) and four microtubule-binding domains (4R) occur in the cerebral cortex (Goedert and Jakes, 1990). Misassembly of the normally unfolded microtubule-associated protein tau into a highly structured amyloid fibril is implicated in the pathological process underlying human tauopathies (Goedert et al., 2017). Alzheimer’s disease (AD), which is primarily associated with 3R and 4R tau (Rosler et al., 2019), is one of the most clinically relevant tauopathies and is the most common neurodegenerative disorder globally, bringing enormous burdens to society and caregivers.

Consequently, molecular imaging of tauopathies has garnered much interest in recent years, and there is increasing recognition of tauopathy as a potential target in the early detection of neurodegenerative disease or indeed as a potential therapeutic target. Among available tracers for positron emission tomography (PET), the selectivity for tau isoforms determines their suitability for particular neurodegenerative disease. For example, a head-to-head comparison of [^11^C]-THK5351 and [^11^C]-PBB3 in AD patients revealed distinct binding patterns for the two tracers in the same patients. [^11^C]-THK5351 binding matched the tau pathology expected for AD, whereas [^11^C]-PBB3 binding showed a greater affiliation with β-amyloid distribution (Chiotis et al., 2018). An immunofluorescence study with PBB3 and AV-1451 both showed intense labeling of non-ghost and ghost tangles, whereas detection of dystrophic neurites in brain of AD patients was clearer for PBB3 (Ono et al., 2017). Further quantitative autoradiographic analysis post-mortem showed moderate [^11^C]-PBB3 autoradiographic binding vs. relatively faint [^18^F]-AV-1451 labeling of the 3R isoforms in brains of patients dying with Pick’s disease (PiD), and likewise for the 4R isoforms in brains of patients dying with progressive supranuclear palsy (PSP) or corticobasal degeneration. However, the binding of the two ligands was similar for paired helical filament (PHF)-tau in patients dying with AD (Ono et al., 2017).

Off-target binding, at present one of the great challenges in molecular neuroimaging of tauopathy, occurs then the tracer has affinity to an unintended molecular target in the brain. For example, the interpretation of tau burden in PET scans with [^18^F]-THK5351 (Ng et al., 2017) and several other ostensibly tau-selective tracers (Murugan et al., 2019) was complicated by off-target binding to monoamine oxidase B (MAO-B). Furthermore, the tau tracers [^18^F]-AV-1451, [^18^F]-THK5351, and [^18^F]-MK6240 all showed additional binding to neuromelanin (Aguero et al., 2019; Tago et al., 2019), which likely accounted for their binding in the midbrain dopamine neurons of the substantia nigra (Marquie et al., 2015; Harada et al., 2016).

PBB3-based tracers show much promise in overcoming the problem of incomplete specificity for tau. Previous studies regarding the prototype [^11^C]-PBB3 revealed no cross-reactivity with monoamine oxidase A (MAO-A) and MAO-B (Ni et al., 2018), although there was a low affinity for non-tau fibrils such as assemblages of amyloid-β (Maruyama et al., 2013; Ono et al., 2017) and α-synuclein aggregates (Koga et al., 2017). Nevertheless, [^11^C]-PBB3 binding was highly selective for tau at the nm radioligand concentrations typically achieved in a human PET study (Koga et al., 2017; Ni et al., 2018). However, routine clinical use of the compound [^11^C]-PBB3 presented logistic difficulties due to the short physical half-life of carbon-11 (Hashimoto et al., 2014, 2015; Shimada et al., 2017). A PBB3 derivative labeled with longer-lived fluorine-18 might overcome this limitation.

Moreover, according to the latest National Institute on Aging – Alzheimer’s Association framework on AD (Jack et al., 2018; Cummings, 2019), a framework comprising three biomarkers β-amyloid (A), tau (T), and neurodegeneration (N) is recommended for defining the AD spectrum and for distinguishing AD from non-AD causes of cognitive impairment. Detection of β-amyloid is accomplished with established PET tracers such as [^11^C]-PiB (Klunk et al., 2004; Jimenez-Bonilla et al., 2016) and [^18^F]-AV45 (Nemmi et al., 2014; Brendel et al., 2015; Lin et al., 2016). These tracers show progressive accumulation of β-amyloid first in isocortical areas and later in limbic and cortical structures. Positron emission tomography with [^18^F]-FDG reveals a characteristic pattern of hypometabolism in temporoparietal cortex and posterior cingulate of AD patients (Minoshima et al., 1995; Kato et al., 2016; Hsu et al., 2017; Rice and Bisdas, 2017; Blazhenets et al., 2019). Multimodal studies combining A/T/N biomarkers showed tau deposition as measured by [^18^F]-AV-1451 (Sintini et al., 2019) or [^18^F]-THK5351 (Baghel et al., 2019) PET, which correlated with hypometabolism to [^18^F]-FDG PET and with atrophy, as measured by structural magnetic resonance imaging (MRI) (Sintini et al., 2019).

In this context, we presented [^18^F]-APN-1607 ([^18^F]-PM-PBB3), a next-generation tau tracer derived from the PBB3 series, but possessing a superior drug metabolism and pharmacokinetic profile, improved specific binding in brain, and the logistic advantage imparted by fluorine-18 (Shimada et al., 2017, 2018; Tagai et al., 2020; see Supplementary Table 1). We aimed in this study to characterize the cerebral uptake pattern of [^18^F]-APN-1607 as a marker for hyperphosphorylated tau in patients with clinically diagnosed AD in comparison to a normal control (NC) group and to investigate the correlation of this regional uptake with hypometabolism to [^18^F]-FDG PET and in relation to impaired cognitive function.

## Materials and Methods

### Subjects

Nineteen clinically diagnosed and amyloid PET-positive AD patients (6 underwent [^11^C]-PiB PET, and 13 underwent [^18^F]-AV45 PET) and 11 NC subjects who also underwent [^18^F]-APN-1607 PET in Huashan Hospital, Shanghai, China, were enrolled in this study from 2018/11 to 2019/11. All subjects underwent anatomical MRI, and all AD patients underwent an [^18^F]-FDG PET within 1 month before or after [^18^F]-APN-1607 PET. The diagnosis of clinically probable AD was based on current diagnostic criteria (McKhann et al., 2011). Experienced radiologists assessed medial temporal lobe atrophy (MTA) using MTA–Visual Rating Scale (VRS) blinded to clinical conditions in all subjects. Experienced neurologists from the cognitive impairment clinic administered the Mini-Mental State Examination (MMSE) and Clinical Dementia Rating (CDR) test for all patients. Meanwhile, the NC group also accepted CDR test. None of the NCs had a history of cognitive impairment, psychiatric illness, central nervous system disease, or head injury. Furthermore, dementia caused by other reasons and mild cognitive impairment (MCI) were excluded after clinical screening by experienced neurologist/cognitive specialists. This study was approved by the ethics committee of Huashan Hospital (no. 2018-363). All procedures performed in this study were in accordance with the ethical standards of the institutional research committee and with the Helsinki Declaration of 1975 and its later amendments. All subjects or a legally responsible relative gave written informed consent before the study.

### Imaging and Processing

#### Radiosynthesis

[^18^F]-APN-1607 was prepared in Huashan Hospital by a nucleophic substitution reaction followed by an acid hydrolysis carried out with an [^18^F]-multifunction synthesizer (Beijing PET Technology Co., Ltd., Beijing, China). APRINOIA Therapeutics (Suzhou, China) provided the tosylate precursor used for the radiosynthesis. After purification with semipreparative high-performance liquid chromatography, the product [^18^F]-APN-1607 was formulated in ascorbate-containing normal saline for injection and was filtered through a sterile membrane filter. The radiosynthesis was completed in 90 min, giving [^18^F]-APN-1607 with a radiochemical purity of ≥90% and a molar activity of ≥37 MBq/μmol at the end of synthesis. The production was conducted under the green light-emitting diode light (510 nm) illumination, and the product was sterile and negative for pyrogens.

#### Image Acquisition and Reconstruction

All subjects were scanned on a Siemens Biograph 64 PET/computed tomography (CT) (Siemens, Erlangen, Germany) in three-dimensional (3D) mode in Huashan Hospital. A low-dose CT transmission scan was performed before PET scanning for attenuation correction. Static emission recordings were acquired during the interval of 90–110 min after intravenous injection of 370 MBq [^18^F]-APN-1607. Image reconstruction was obtained by the ordered subset expectation maximization 3D (OSEM 3D) method. Patients with AD underwent [^18^F]-FDG PET on another scanning day with intravenous injection of 185 MBq, following a scanning procedure and OSEM 3D reconstruction as described in a previous study (Wu et al., 2013). All subjects also underwent anatomical MRI in a 3.0-T horizontal magnet (Discovery MR750; GE Medical Systems, Milwaukee, WI, United States) at Huashan Hospital.

#### Semiquantitative Volume of Interest–Based PET Analyses

The PNEURO data processing pipeline of PMOD version 4.005 (PMOD Technologies Ltd., Zurich, Switzerland) was used for spatial normalization of all PET images to the Montreal Neurological Institute (MNI) space, using the individual MRI as an intermediate. Both [^18^F]-APN-1607 and [^18^F]-FDG PET images were analyzed in the following manner: we first segmented the individual MRI into gray matter (GM), white matter (WM), and cerebrospinal fluid (CSF) and made the spatial normalization to the MNI space. Subsequently, each subject’s individual PET images were spatially matched to MRI and then resampled using the normalization arising from the GM/WM/CSF MRI segmentation procedure (Ashburner, 2007). Based on the Atlas template [adult brain maximum probability map (“Hammersmith atlas”; n30r83)], the whole brain was parcellated into the following regions for standardized uptake value ratio (SUVR) calculations: frontal, temporal, occipital, and parietal lobes; insula; cingulum; caudate; putamen; pallidum; thalamus; midbrain; pons; medulla; and cerebellar cortex. The cerebellar cortex was selected as the reference region for tau images because it has negligible tau pathology to examination post-mortem AD cerebellum (Herrmann et al., 1999; Baghel et al., 2019). The same reference region was used for [^18^F]-FDG PET images (Leuzy et al., 2018; Baghel et al., 2019).

#### Voxel-Wise Analyses

Statistical parametric mapping (SPM) analysis was performed using SPM8 (Wellcome Department of Cognitive Neurology, London, United Kingdom) implemented in MATLAB 8.4 (R2014) (Mathworks Inc., Sherborn, MA, United States). The [^18^F]-APN-1607 SUVR maps from the AD and NC groups were compared by a voxel-wise two-tailed Student *t*-test after 10-mm Gaussian smoothing. To evaluate significant differences, we set the voxel threshold at *P* < 0.01 [familywise error (FWE)-corrected] over the whole brain with an extent threshold empirically chosen to be at least twice of the expected number of voxels per cluster estimated in the SPM run. Significant regions were localized by Talairach–Daemon software (Research Imaging Center, University of Texas Health Science Center, San Antonio, TX, United States). The SPM maps for abnormal [^18^F]-APN-1607 uptakes were overlaid on a standard structural MRI brain template in stereotaxic space. We then used multiple regression analyses to determine the relationship between [^18^F]-APN-1607 uptake values and MMSE in the AD patients. Voxels surviving *P* < 0.01 (uncorrected) with an extent threshold of at least twice of the expected number of voxels per cluster estimated in the SPM run were considered significant for the multiple regression analyses. Moreover, clusters surviving at FWE *P* < 0.05 were also searched for these multiple regression analyses.

### Statistical Analysis

Demographic characteristics and semiquantitative PET results in different target volumes of interest (VOIs) were compared between AD and NC groups using the independent two-tailed Student *t*-test, χ^2^ test, or Mann-Whitney *U* test as appropriate. Effect sizes for the discrimination between patients and NC subjects were evaluated by Cohen *d*. Correlation analyses between PET SUVR in target VOIs and clinical parameter (MMSE), as well as intermodality correlations, were performed using Spearman correlation. All statistical analyses were performed in SPSS version 22.0 software (SPSS Inc., Chicago, IL, United States). *P* < 0.05 was considered significant.

## Results

### Demographic Information and Clinical Characteristics

Our AD group consisted of 7 males and 12 females with mean age 61.8 (±11.3) years, whereas NC group included seven males and four females with mean age 61.8years (±4.6) years. There were 14 early onset AD, who showed symptoms younger than 65 years old and five late-onset AD (LOAD) in our cohort. Both groups were comparable for age of scanning (*P* = 0.992) and gender (*P* = 0.156). The general cognition of the AD group as assessed by CDR-Global Score (CDR-GS) and MMSE score showed that our patient cohort was mainly at moderate to advanced stages of the disease (CDR-GS: 1 (1–2); MMSE: 17.0 ± 7.6). The MTA-VRS showed that AD group had abnormal brain atrophy while NC group showed normal (Table 1).

**TABLE 1:** Demographic information and clinical characteristics.

Group	No.	Gender (male/female)	Age of scanning (y)	Age at onset (y)	Education (y)	MMSE score	CDR-GS	MTA-VRS
AD	19	7/12	61.8 ± 11.3	56.9 ± 10.5	10.7 ± 4.2	17.0 ± 7.6	1 (1–2)	2 (2–2)
NC	11	7/4	61.8 ± 4.6	–	N.A.	N.A.	0 (0–0)	1 (0–1)
*P*	–	0.156^a^	0.992^b^	–	–	–	<0.001^c^	<0.001^c^

**For age, education years, and MMSE score; the expression means mean ± standard deviation. For CDR-GS and MTA-VRS, the expression means median (interquartile range). ^*a*^χ^2^ test was performed. ^*b*^Independent two-tailed Student t test was performed. ^*c*^Mann-Whitney U test was performed. AD, Alzheimer’s disease; NC, normal control; MMSE, Mini-Mental State Examination; CDR-GS, Clinical Dementia Rating Global Score; MTA-VRS, Medial Temporal Lobe Atrophy Visual Rating Scale; N.A., not available; y, year.**

### Semiquantitative VOI-Based PET Analyses

**FIGURE 1:**
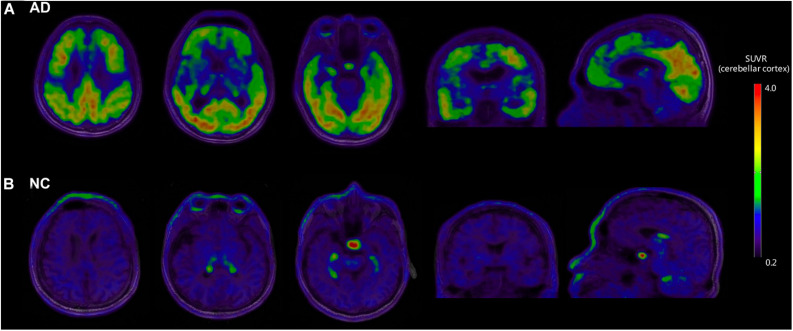
Representative examples of [^18^F]-APN-1607 PET and anatomical MRI superimposed images of AD and NC subjects. **(A)** [^18^F]-APN-1607 PET of an AD patient (male, 56 years old, MMSE 17, 11 years’ education experience, memory impairment complaint for 5 years with positive [^11^C]-PiB result). **(B)** [^18^F]-APN-1607 PET of a NC subject (Male, 61y). AD, Alzheimer’s disease; NC, normal control. The color stripe indicates the standard uptake value ratio with cerebellar cortex as the reference region.

Figure 1 shows the representative examples of [^18^F]-APN-1607 PET and anatomical MRI superimposed images of AD and NC subjects.

**FIGURE 2:**
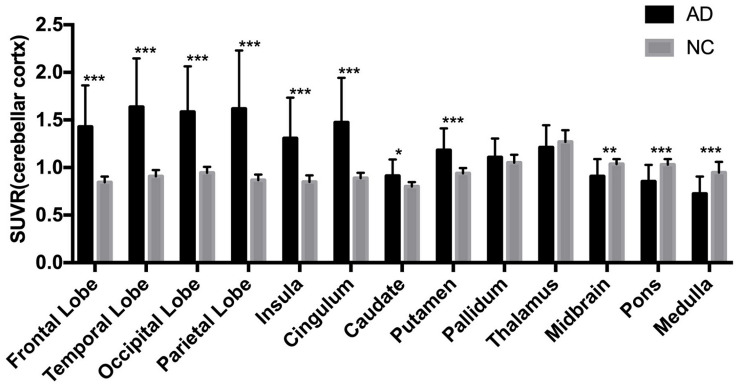
Differences of regional [^18^F]-APN-1607 SUVR between AD and NC groups. AD, Alzheimer’s disease; NC, normal control. *P < 0.05, **P < 0.01, ***P < 0.001.

The AD group showed abnormally higher [^18^F]-APN-1607 binding than did the NC group in all cerebral lobes (*P* < 0.001, Cohen *d* varying from 1.5 to 2.1), as well as in caudate (*P* < 0.05, Cohen *d* = 0.9) and putamen (*P* < 0.001, Cohen *d* = 1.5). Meanwhile, the AD group showed lower uptake of [^18^F]-APN-1607 in midbrain (*P* < 0.01, Cohen *d* = 1.0), pons (*P* < 0.001, Cohen *d* = 1.4), and medulla (*P* < 0.001, Cohen *d* = 1.5) (Table 2 and Figure 2).

**TABLE 2:** Differences of [^18^F]-APN-1607 regional SUVR between AD and NC groups.

VOI	SUVR		*P*	Cohen *d*
	AD	NC		
Frontal lobe	1.43 ± 0.42	0.85 ± 0.06	< 0.001***	1.926
Temporal lobe	1.64 ± 0.50	0.91 ± 0.06	< 0.001***	2.062
Occipital lobe	1.58 ± 0.47	0.95 ± 0.06	< 0.001***	1.917
Parietal lobe	1.62 ± 0.60	0.87 ± 0.06	< 0.001***	1.773
Insula	1.31 ± 0.41	0.85 ± 0.07	< 0.001***	1.546
Cingulum	1.47 ± 0.46	0.89 ± 0.05	< 0.001***	1.801
Caudate	0.91 ± 0.17	0.80 ± 0.04	0.015*	0.898
Putamen	1.18 ± 0.22	0.94 ± 0.05	< 0.001***	1.508
Pallidum	1.11 ± 0.19	1.05 ± 0.08	0.295	0.376
Thalamus	1.21 ± 0.23	1.27 ± 0.12	0.374	0.325
Midbrain	0.91 ± 0.17	1.04 ± 0.05	0.008**	1.005
Pons	0.85 ± 0.17	1.03 ± 0.06	< 0.001***	1.397
Medulla	0.72 ± 0.18	0.95 ± 0.11	< 0.001***	1.537

For SUVR, the expression means mean ± standard deviation. VOI, volume of interest; SUVR, standard uptake value ratio; AD, Alzheimer’s disease; NC, normal control. *P < 0.05, **P < 0.01, ***P < 0.001.

As for clinical associations, [^18^F]-APN-1607 binding in all cerebral lobes (with the exception of the occipital lobe) correlated negatively with the MMSE score (frontal lobe: *R* = −0.632, *P* = 0.004; temporal lobe: *R* = −0.593, *P* = 0.008; parietal lobe: *R* = −0.552, *P* = 0.014; insula: *R* = −0.650, *P* = 0*P* = 0.003; cingulum: *R* = −0.665, *P* = 0.002). Likewise, [^18^F]-APN-1607 binding in putamen, thalamus and medulla showed similar correlations (putamen: *R* = −0.557, *P* = 0.013; thalamus: *R* = −0.595, *P* = 0.007; medulla: *R* = −0.469, *P* = 0.043). [^18^F]-FDG uptake, as a surrogate marker for neuronal metabolic activity, showed positive correlations with MMSE in the frontal and parietal lobes (frontal lobe: *R* = 0.469, *P* = 0.043, parietal lobe: *R* = 0.550, *P* = 0.015). The temporal lobe also showed a broadly similar correlation with MMSE, albeit without reaching significance (*R* = 0.444, *P* = 0.057) (Table 3).

**TABLE 3:** Correlations between regional SUVR and MMSE score, and correlations between multimodes in AD group.

VOI	^18^F-APN-1607 and MMSE		^18^F-FDG and MMSE		^18^F-APN-1607 and ^18^F-FDG	
	*P*	*R*	*P*	*R*	*P*	*R*
Frontal lobe	0.004**	–0.632	0.043*	0.469	0.073	–0.421
Temporal lobe	0.008**	–0.593	0.057	0.444	0.020*	–0.530
Occipital lobe	0.076	–0.417	0.341	0.231	0.011*	–0.567
Parietal lobe	0.014*	–0.552	0.015*	0.550	0.003**	–0.637
Insula	0.003**	–0.650	0.411	0.200	0.616	–0.123
Cingulum	0.002**	–0.665	0.630	0.118	0.333	–0.235
Caudate	0.067	–0.429	0.780	–0.069	0.091	0.398
Putamen	0.013*	–0.557	0.693	–0.097	0.251	0.277
Pallidum	0.395	–0.207	0.102	–0.387	0.743	0.081
Thalamus	0.007**	–0.595	0.912	0.027	0.808	–0.060
Midbrain	0.531	–0.153	0.150	–0.343	0.892	–0.033
Pons	0.301	–0.250	0.461	–0.180	0.399	–0.205
Medulla	0.043*	–0.469	0.080	–0.411	0.705	0.093

VOI, volume of interest; R, Spearman correlation. *P < 0.05, **P < 0.01, ***P < 0.001.

We then interrogated the relationships between [^18^F]-APN-1607 and [^18^F]-FDG. We found negative correlations between tau deposition and FDG uptake in the temporal, parietal, and occipital lobes (temporal lobe: *R* = −0.530, *P* = 0.020; parietal lobe: *R* = −0.637, *P* = 0.003; occipital lobe: *R* = −0.567, *P* = 0.011). A similar pattern was observed for the frontal lobe, albeit without reaching statistical significance (*R* = −0.421, *P* = 0.073) (Table 3).

### Voxel-Wise PET Analyses

**FIGURE 3:**
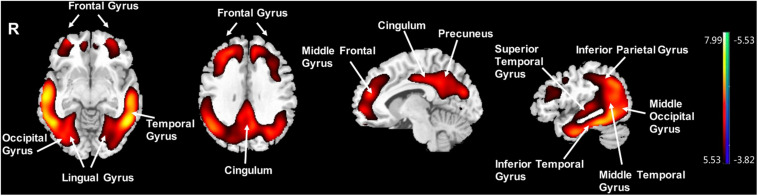
Voxel-wise differences of whole-brain [^18^F]-APN-1607 binding in the AD group compared to the NC Group. AD, Alzheimer’s disease; NC, normal control. Increased binding is displayed in red; decreased binding is displayed in blue. The color stripe indicates the *t* value; voxel threshold *P* < 0.01, FWE-corrected. *T* statistic maps are displayed in MNI standard space.

Compared to the NC group, the AD group had elevated [^18^F]-APN-1607 binding in fusiform gyrus (BA 37), superior temporal gyrus (BA 21), inferior temporal gyrus (BA 20), middle frontal gyrus (BA 8, 9, 11), and cingulate gyrus (BA 32) at FWE *P* < 0.01 (Table 4 and Figure 3).

**TABLE 4:** Brain regions with significant increased uptakes of [^18^F]-APN-1607 in AD group compared to NC Group (*P* < 0.01, FWE corrected).

Region*	Hemisphere	Cluster size (mm^3^)	Z max	Coordinates^†^		
				X	Y	Z
Fusiform gyrus (BA 37)	Left	283,064	5.72	−48	−44	−18
Superior temporal gyrus (BA 21)	Right	283,064	5.53	62	−22	−4
Inferior temporal gyrus (BA 20)	Left	283,064	5.50	−54	−24	−22
Middle frontal gyrus (BA 8)	Right	56,784	5.17	22	38	34
Middle frontal gyrus (BA 8)	Right	56,784	5.17	30	30	34
Middle frontal gyrus (BA 9)	Right	56,784	5.13	10	48	18
Middle frontal gyrus (BA 8)	Left	51,360	5.06	−10	38	34
Middle frontal gyrus (BA 11)	Left	51,360	5.05	−26	48	−14
Cingulate gyrus (BA 12)	Left	51,360	5.03	−10	26	42

*Significant at FWE-corrected voxel threshold of P < 0.01, extent threshold = 58 voxels (464 mm^3^). ^†^Coordinates are displayed in MNI standard space. BA, Brodmann area; FWE, familywise error.

**FIGURE 4:**
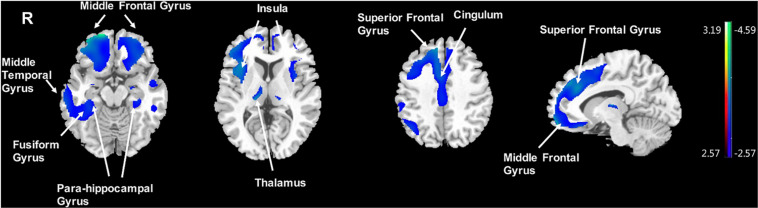
Voxel-wise correlation of MMSE score and whole-brain [^18^F]-APN-1607 binding in the AD group. AD, Alzheimer’s disease; NC, normal control. Positive correlations are displayed in red; negative correlations are displayed in blue. The color stripe indicates the *t* value; voxel threshold *P* < 0.01, uncorrected. Images are displayed in MNI standard space.

Significantly negative correlations between MMSE and brain [^18^F]-APN-1607 binding were found mainly in superior frontal gyrus (BA 10), middle frontal gyrus (BA 9, 10, 11, 47), parahippocampal gyrus (BA 36) and lateral globus pallidus at *P* < 0.01 (uncorrected). Of these, both the superior frontal gyrus (BA 10) and middle frontal gyrus (BA 9, 10) survived FWE at *P* < 0.05 (Table 5 and Figure 4).

**TABLE 5:** Brain regions exhibiting a significant negative correlation between MMSE score and regional brain uptakes of [^18^F]-APN-1607 in AD group (*P* < 0.01, uncorrected).

Region*	Hemisphere	Cluster size (mm^3^)	*Z* Max	Coordinates^†^		
				X	Y	Z
Superior frontal gyrus (BA 10)^‡^	Right	91,784	3.65	16	60	−16
Middle frontal gyrus (BA 10)^‡^	Right	91,784	3.43	36	52	−16
Middle frontal gyrus (BA 9)^‡^	Right	91,784	3.24	38	36	28
Middle temporal gyrus (BA 21)	Right	19,376	3.00	68	−28	−10
Parahippocampal gyrus (BA 36)	Right	19,376	2.95	34	−34	−12
Lentiform nucleus (lateral globus pallidus)	Right	19,376	2.89	28	−12	−12
Middle frontal gyrus (BA 10)	Left	20,664	2.98	−30	54	−16
Middle frontal gyrus (BA 47)	Left	20,664	2.90	−46	38	−10
Middle frontal gyrus (BA 11)	Left	20,664	2.87	−26	34	−20

*Significant at uncorrected voxel threshold of P < 0.01, extent threshold = 937 voxels (7,504 mm^3^). ^†^Coordinates are displayed in MNI standard space. ^‡^Survived at FWE-corrected voxel threshold of P < 0.05. BA, Brodmann area; FWE, familywise error.

## Discussion

In this present study, we characterized the [^18^F]-APN-1607 uptake pattern in patients with AD in comparison with NC subjects. Furthermore, we assessed the correlation with between tau PET with [^18^F]-FDG PET and clinical parameters (MMSE). Our main objective was to characterize the utility of this next-generation tau tracer in terms of effect size for detecting disease-specific tau deposition, thus confirming the known relationship between tau deposition and decreased FDG uptake and cognitive impairment in AD patients.

In the regional analysis of [^18^F]-APN-1607, we found AD patients had higher binding than NC subjects in widespread regions of the cerebral cortex, caudate, and putamen and conversely relatively low uptake in the brainstem. The voxel-wise analysis results were in agreement with those of the region-based analysis, and the cerebral regions with increased tau deposition to [^18^F]-APN-1607 were consistent with previously published studies for previous-generation tau tracers (Table 6).

**TABLE 6:** Summary of ROI analyses of different tau tracers in AD and NC groups.

	Cohorts		NC	AD	Cohen *d*
[^18^F]-AV-1451 (Shcherbinin et al., 2016)	AD *N* = 5, MMSE = 21.8 (4.1) NC *N* = 5, MMSE = MMSE = 29.6 (0.5)	Frontal	1.00 (0.16)	1.65 (0.69)	1.30
		Occipital	1.07 (0.18)	1.67 (0.59)	1.38
		Lateral parietal	1.05 (0.19)	2.03 (0.84)	1.61
		Mesial temporal	1.09 (0.14)	1.78 (0.48)	1.95
		Lateral temporal	1.10 (0.15)	2.15 (0.89)	1.65
		Putamen	1.34 (0.09)	1.78 (0.23)	2.52
[^18^F]-THK5351 (Kang et al., 2017)	AD *N* = 51, MMSE = 13.8 (6.0) NC *N* = 43, MMSE = 28.5 (1.6)	Frontal	1.35 (0.22)	2.05 (0.34)	2.44
		Occipital	1.09 (0.15)	1.57 (0.46)	1.40
		Superior parietal	1.18 (0.20)	1.80 (0.43)	1.85
		Inferior parietal	1.34 (0.21)	2.60 (0.69)	2.47
		Lateral temporal	1.60 (0.23)	2.71 (0.49)	2.90
		Mesial temporal	2.42 (0.27)	3.52 (0.59)	2.40
		Anterior cingulate	2.88 (0.43)	3.40 (0.57)	1.03
		Hippocampus	2.44 (0.27)	3.40 (0.55)	2.22
		Fusiform	1.51 (0.19)	2.39 (0.57)	2.07
		Entorhinal	2.15 (0.34)	3.36 (0.89)	1.80
		Basal ganglia	3.08 (0.48)	3.72 (0.62)	1.15
[^11^C]-PBB3 (Kitamura et al., 2018)	AD/MCI *N* = 7, MMSE = 23.7 (6.9) NC *N* = 9, MMSE = 29.4 (0.7)	Frontal	0.86 (0.04)	0.94 (0.09)	1.15
		Occipital	0.90 (0.03)	1.05 (0.07)	2.79
		Parietal	0.83 (0.05)	0.97 (0.09)	1.92
		Medial temporal	0.99 (0.05)	1.02 (0.05)	0.60
		Lateral temporal	0.95 (0.04)	1.05 (0.06)	1.96
		Anterior cingulate	0.91 (0.07)	0.94 (0.01)	0.60
[^18^F]-MK6240 (Lohith et al., 2019)	AD *N* = 4, MMSE = 16.5 (7.3) NC *N* = 4, MMSE = 29.0 (0.0)	Temporal	0.98 (0.07)	1.64 (0.72)	1.29
		Hippocampus	0.93 (0.10)	1.37 (0.25)	2.31
		Amygdala	0.84 (0.11)	1.67 (0.40)	2.83
		Caudate	0.79 (0.06)	0.71 (0.16)	0.66
		Putamen	0.91 (0.04)	1.15 (0.35)	0.96
[^18^F]-RO948 (Wong et al., 2018)	AD *N* = 11, MMSE = 20.8 (2.7) NC *N* = 5)	Middle frontal lobe	1.13 (0.10)	2.27 (1.40)	1.15
		Lateral occipital lobe	1.35 (0.10)	2.34 (1.30)	1.07
		Precuneus	1.16 (0.20)	2.29 (1.50)	1.06
		Superior parietal	1.24 (0.10)	2.18 (1.10)	1.20
		Inferior parietal	1.32 (0.10)	2.8 (1.50)	1.39
		Inferior temporal	1.14 (0.10)	2.75 (1.40)	1.62
		Middle temporal	1.15 (0.10)	2.54 (1.40)	1.40
		Posterior cingulate	1.1 (0.10)	1.96 (1.20)	1.01
		Insula	0.98 (0.10)	1.46 (0.50)	1.33
		Hippocampus	0.90 (0.20)	1.26 (0.30)	1.40
		Parahippocampus	0.96 (0.10)	1.95 (0.70)	1.97
		Amygdala	0.81 (0.10)	1.72 (0.70)	1.83
		Caudate	0.80 (0.10)	0.97 (0.20)	1.06
		Putamen	0.93 (0.10)	1.28 (0.20)	2.24
		Thalamus	0.90 (0.10)	1.00 (0.20)	0.69
[^18^F]-PI2620 (Mueller et al., 2019)	AD *N* = 12, MMSE = 20.4 (6.3) NC *N* = 10, MMSE = 29.0 (1.2)	Occipital	1.08 (0.08)	1.45 (0.23)	2.15
		Parietal	1.03 (0.06)	1.53 (0.40)	1.75
		Superior temporal	1.07 (0.10)	1.36 (0.23)	1.64
		Inferior temporal	1.09 (0.10)	1.80 (0.40)	2.44
		Posterior cingulate	1.03 (0.13)	1.82 (0.68)	1.61
		Hippocampus	1.07 (0.08)	1.26 (0.20)	1.25
		Parahippocampus	1.07 (0.11)	1.46 (0.29)	1.78
		Amygdala	0.99 (0.11)	1.29 (0.29)	1.37
		Fusiform	1.07 (0.11)	1.63 (0.29)	2.55
		Striatum	0.83 (0.1)	1.02 (0.14)	1.56
		Pallidum	0.95 (0.17)	1.10 (0.14)	0.96
		Thalamus	0.93 (0.11)	0.96 (0.07)	0.33

*AD, Alzheimer’s disease; NC, normal control.*

Hitherto, only limited data for second-generation tau tracers have been reported (Leuzy et al., 2019). Some novel tau tracers generally presented a number of advantages over the previous generation, notably with respect to target selectivity. For example, *in vitro* studies suggested that [^18^F]-MK6240 and [^18^F]-JNJ-067 bound with neurofibrillary tangle (NFT) (Hostetler et al., 2016), whereas [^18^F]-RO948 bound to both NFT and neuropil threads (Honer et al., 2018), whereas [^18^F]-JNJ64349311 interacted with PHF-tau and neuropil threads (Declercq et al., 2017). [^18^F]-PI2620 showed ambivalent binding to 3R-tau from PiD brain and to 4R-tau from PSP samples (Kroth et al., 2019). [^18^F]-APN-1607 showed specific binding with tau aggregates in AD and PSP (Shimada et al., 2018). Furthermore, such tracers showed improvement in off-target binding. [^18^F]-APN-1607 showed no off-target binding in the basal ganglia and thalamus (Shimada et al., 2017), and [^18^F]-APN-1607, [^18^F]-MK6240, [^18^F]-JNJ-607, and [^18^F]-PI2620 revealed no off-target binding with MAO-A and MAO-B. Extensive screening of [^18^F]-RO948 also revealed no off-target binding. These limited available data suggested that novel-generation tau tracers had significant advantages in comparison to previous generation tracers. The selectivity for tau isoforms of the different series renders them particularly suitable for the investigation of neurodegenerative disease apparent. Further clinical studies are required to demonstrate their diagnostic utility and to further evaluate their performance.

The limited *in vivo* data reported for other new-generation tau tracers showed similar results to our study: [^18^F]-MK6240, [^18^F]RO-948, and [^18^F]-PI6260 showed elevated signals in temporal areas and more broadly throughout the cortex in AD patients compared to NC subjects. The SUVRs in temporal lobe of AD/MCI patients were 1.64 (±0.72) with [^18^F]-MK6240 [MMSE = 18.8 (±6.9), Cohen *d* = 1.3) (Lohith et al., 2019), 2.75 (±1.40) with [^18^F]RO-948 (inferior temporal lobe, MMSE = 20.8 (±2.7), Cohen *d* = 1.6] (Wong et al., 2018), and 1.80 (±0.40) with [^18^F]-PI2620 [inferior temporal, MMSE = 20.4 (±6.3), Cohen *d* = 2.4] (Mueller et al., 2019). Our findings of [^18^F]-APN-1607 had relatively lower SUVR in the present study [temporal lobe: 1.64 (±0.50), MMSE = 17.0 (±7.6), Cohen *d* = 2.1] comparing to [^18^F]RO-948 and [^18^F]-PI2620, which might arise from the relatively rough ROI; however, the relatively high Cohen *d* value indicated that [^18^F]-APN-1607 was a highly sensitive tracer for detecting tau aggregates in AD. Findings in other brain regions were also comparable among the next-generation tau tracers, including [^18^F]-APN-1607 (Table 6).

In addition to the finding of distinctly elevated [^18^F]-APN-1607 binding in the widespread cerebral cortex of patients with AD of moderate severity, we also saw increased signals in the striatum. We noted that [^18^F]-AV-1451, [^18^F]-RO948, and [^18^F]-PI2620 likewise showed similarly increased signals in the striatum (Shcherbinin et al., 2016; Wong et al., 2018; Mueller et al., 2019). Previous neuropathological observation addressed that NFT occurs in striatum in late Braak stages of AD (V and VI) (Chan and Shea, 2006). Another autopsied study reinforced AD cases with severe putaminal tauopathy at the advanced stages and indicated that severe microtubule-associated protein tau accumulation in the basal ganglia might occur most frequently in AD cases, without comorbidity of other neurodegenerative diseases in a general aging population (Hamasaki et al., 2019). Accordingly, the higher [^18^F]-APN-1607 PET signal seen in caudate and putamen of our AD group might suggest higher than expected tauopathy (Su et al., 2015). Nonetheless, previous MRI studies reported iron overloading in striatum in AD (Bartzokis et al., 1993, 1994; Acosta-Cabronero et al., 2013). Iron is a known component of neuritic plaques (Grundke-Iqbal et al., 1990; Connor et al., 1992; LeVine, 1997; Lovell et al., 1998) and neurofibrillary tangles (Good et al., 1992), and the Fenton reaction has long been suspected to contribute to AD pathology (Smith et al., 1997). Indeed, excessive ferrous iron may well favor β-amyloid aggregation and otherwise produce neurotoxicity (Schubert and Chevion, 1995; Leskovjan et al., 2011). Furthermore, iron can induce hyperphosphorylation and aggregation of tau (Lovell et al., 2004; Chan and Shea, 2006). Altogether, we interpreted these findings with much caution, given that previous generation tau tracers such as [^18^F]-AV-1451 demonstrated off-target binding to ferrous iron (Lowe et al., 2016; Passamonti et al., 2017; Choi et al., 2018; Baker et al., 2019), which as described above was implicated in the neuropathology of neurofibrillary tangle formation. Noteworthy, [^18^F]-AV-1451 has shown non-specific binding in the striatum in healthy elderly subjects (Marquie et al., 2015; Passamonti et al., 2017; Smith et al., 2017), and its binding appeared to increase with healthy aging (Smith et al., 2017). Similarly, the first-generation [^11^C]-PBB3 also showed off-target binding in the basal ganglia, the reason for which was yet to be determined (Ono et al., 2017), although a recent abstract indicated there was no off-target binding in the basal ganglia and thalamus for [^18^F]-APN-1607 (Shimada et al., 2017).

In this study, we investigated clinical correlations for [^18^F]-APN-1607 and [^18^F]-FDG uptakes separately. The MMSE score, as a surrogate for general cognition, showed strong correlations with [^18^F]-APN-1607 SUVR in all cerebral cortical regions except the occipital lobe, and with [^18^F]-FDG SUVR in the frontal and parietal lobes (Table 3). In [^18^F]-APN-1607, we also saw relatively weaker correlations in the putamen, thalamus, and medulla.

It was hypothesized that accumulation of toxic intracellular aggregate accompanied by a loss of soluble tau capable of stabilizing microtubules might synergistically lead to compromised neuronal survival (Lee et al., 2011), accounting for the putative relationship between NFT burden and cognitive decline in AD patients (Terry et al., 1981; Gomez-Isla et al., 1997). According to the Braak theory of AD propagation (Braak and Braak, 1991b; Braak and Del Tredici, 2011), tau deposition spreads widely and aggregates in neocortical areas in advanced AD patients. The association we observed between tau depositions to [^18^F]-PAPN-1607 and general cognitive impairment was consistent with these known relationships, which we could now confirm here with a new-generation tau tracer. The occipital lobe was thought to be affected at only a late stage of typical AD progression (Delacourte et al., 1999) and likewise showed tau accumulation only at a late stage (Alafuzoff et al., 2008). In this regard, our study’s failure to show a correlation between tau deposition and [^18^F]-APN-1607 in the occipital lobe, and MMSE score was consistent with the moderate severity of disease in our patient group.

We found a negative correlation between tau deposition to [^18^F]-APN-1607 in some subcortical regions and MMSE score. Degeneration of deep gray matter structures other than the hippocampus and the amygdala occurred in the process of AD and ultimately contributes to cognitive deterioration. The thalamus pathology played an increasingly recognized role in early memory loss of AD patients (Aggleton and Brown, 1999; Harding et al., 2000; Taber et al., 2004; Carlesimo et al., 2011). The anterodorsal thalamic nucleus was posited to undergo neurofibrillary changes concomitant with the hippocampus (Braak Stages III–IV) (Braak and Braak, 1991b). Nonetheless, some thalamic nuclei appeared unaffected or show only mild neurofibrillary changes, even in cases of severe AD (Braak and Braak, 1991b). Recent studies have provided evidence that AD-related tau cytoskeletal pathology is initiated in subcortical regions, which supports the widely held hypothesis that early occurring subcortical tau cytoskeletal pathology, including that in the thalamus, may play a crucial role in the cascade of the early pathological events of AD (Rub et al., 2000; Grinberg et al., 2009; Stratmann et al., 2016). In addition, the clinical manifestation and progression of AD correlated with loss of neurons, synaptic degeneration in the neocortex and the topographical distribution of the tau cytoskeletal pathology in diseased brains (Jack et al., 2016). The role of the cortical and subcortical regions of the limbic system in the performance of normal cognitive and memory functions was well-known. The presence of severe tau cytoskeletal pathology in the thalamic nuclei with limbic connectivity likely contributed to impaired neural processing in limbic circuits, manifesting in certain cardinal symptoms of AD (Braak and Braak, 1991a, b; Rub et al., 2000, 2002; Blennow et al., 2006; Lace et al., 2007; Grinberg et al., 2009; Braak and Del Trecidi, 2015). Structural MRI studies also showed that overall thalamic volume correlates with cognitive status in MCI and AD patients (de Jong et al., 2008; Pedro et al., 2012; Yi et al., 2016), further highlighting its role in the pathogenesis of AD. In this regard, the negative correlation between [^18^F]-APN-1607 binding in the thalamus and MMSE score in the AD group offered further support, a link between thalamic pathology and cognitive impairment. Like thalamus, the basal nuclei participated in many different neuronal pathways, with functions extending to emotional, motivational, associative, and cognitive processes (Herrero et al., 2002). A previous MRI study has found significantly reduced putamen volumes in AD patients, correlating with impaired cognition (de Jong et al., 2008). Combined with the neuropathological observation that NFT occurs in striatum in Braak stages V and VI in AD (Chan and Shea, 2006), our findings strongly suggested that the tau deposition in the subcortical regions may also contribute to cognitive decline in AD.

Cerebral [^18^F]-FDG uptake provides a surrogate marker for neuronal metabolism. [^18^F]-FDG uptake in the frontal (*R* = 0.469, *P* = 0.043) and parietal lobes (*R* = 0.550, *P* = 0.015) correlated with MMSE score of our AD group, with weaker association for the temporal lobe (*R* = 0.444, *P* = 0.057). Numerous PET studies have demonstrated hypometabolism in the temporoparietal cortex of AD patients (Minoshima et al., 1995; Kato et al., 2016; Rice and Bisdas, 2017), whereas hypometabolism in the frontal lobe is often observed during the progression of AD dementia (Herholz et al., 2007). Thus, our present findings of regional correlation were concordant with the known hypometabolic regions in AD. The relatively weak correlation in the temporal lobe could be indicative of involvement at an early stage of neurodegeneration, with stabilization of the hypometabolism late in this disease, resulting in a weakening of the relationship between temporal hypometabolism with cognitive scores. Indeed, previous [^18^F]-FDG PET studies have suggested that temporal hypometabolism peaks at the MCI stage and does not progress at later stages of the disease (Dukart et al., 2013), which seems consistent with present findings.

Our multimodal analysis revealed several brain regions showing correlations between uptake of both tracers. Previous studies posited that cognitive deficits in AD could arise directly from both tau pathology and subsequent downstream neurodegeneration (Bejanin et al., 2017). We found that SUVRs in the temporal (*R* = −0.530, *P* = 0.020), parietal (*R* = −0.637, *P* = 0.003), and occipital (*R* = −0.567, *P* = 0.011) lobes correlated negatively between [^18^F]-APN-1607 and [^18^F]-FDG uptakes, with a trend toward negative correlation in the frontal lobe (*P* = 0.073, *R* = −0.421). These findings were consistent with results disclosed using other tau tracers [[^18^F]-AV-1451 (Gordon et al., 2019; Sintini et al., 2019) and [^18^F]-THK5351 (Baghel et al., 2019)]. Previous stereological and non-stereological quantitative post-mortem studies of cerebral cortex (Terry et al., 1981, 1987; Cras et al., 1995; Schwab et al., 1998, 1999; Bussiere et al., 2002; Kril et al., 2002) and clinicopathologic studies (Giannakopoulos et al., 2003) reported a close association between NFT counts and neuron loss. However, non–NFT-related mechanisms of neurodegeneration may also play a role in the loss of cortical neurons in AD (Gomez-Isla et al., 1997).

In summary, this preliminary clinical study confirmed the utility of the new compound [^18^F]-APN-1607 for the detection of tau pathology and reinforced previous studies showing an overlap between cerebral glucose hypometabolism (using [^18^F]-FDG PET) and tau deposition and their association with dementia.

### Limitations

We noted several shortcomings in this study. First, our group size in this preliminary study was relatively small. Second, to reduce the radiation exposure to the NC group, we did not perform additional β-amyloid or [^18^F]-FDG PET scanning to conform lack of pathology. However, clinical and MRI assessment revealed normal results. Although the abnormal metabolic regions of AD patients are well-described from a plethora of previous publications, we lacked [^18^F]-FDG PET data for the NC group. In mitigation, we restricted our exploration in the AD group with region-to-region analysis, aiming to explore anatomic relationships between tau deposition and abnormal metabolism. We draw attention to the fact that MMSE reflected only general cognition, whereas individuals may suffer from cognitive impairment in different domains. Future studies involving more detailed cognitive assessments would support a better exploration of the relationships between tauopathy and cognition.

We were cognizant of the methodological challenges associated with choice of reference region. We used cerebellum, a commonly chosen region, for normalization of [^18^F]-APN-1607 and [^18^F]-FDG scans to SUVR maps. Validity of this approach naturally required absence of important pathologies in cerebellum. In future studies, we shall consider other possible methods of normalization. Finally, clinical studies of prospective design are required to further validate the promising results reported here.

## Data Availability Statement

The raw data supporting the conclusions of this article will be made available by the authors, without undue reservation, to any qualified researcher.

## Ethics Statement

The studies involving human participants were reviewed and approved by the Institutional Review Board of Huashan Hospital (HIRB), Fudan University. The patients/participants provided their written informed consent to participate in this study.

## Author Contributions

QZ, YG, CZ, and AR: research program conception. QZ, YG, CZ, JL, and WB: research program organization. JL, WB, ML, LL, ZZ, HL, and ZX: research program execution. CZ, AR, PC, and JL: statistical analyses design. JL, WB, and LL: statistical analyses execution. CZ, AR, QZ, and YG: statistical analyses review and critique. JL and IA: manuscript writing of the first draft. QZ, YG, MB, PC, CZ, and AR: manuscript review and critique. All authors contributed to the article and approved the submitted version.

## Conflict of Interest

The authors declare that the research was conducted in the absence of any commercial or financial relationships that could be construed as a potential conflict of interest.
