# Supplementary material for: Associations of [18F]-APN-1607 Tau PET Binding in the Brain of Alzheimer’s Disease Patients With Cognition and Glucose Metabolism
Source: Front Neurosci. 2020 Jun 30;14:604. doi: 10.3389/fnins.2020.00604 (PMC7338611; doi:10.3389/fnins.2020.00604)
Supplement: Supplementary file 1 [file Table_1.DOCX]

Supplementary Material

# Supplementary Table

Table 1s Chemical structures and in vitro characteristics of first- and second-generation tau PET tracers.

| First-generation tau PET tracer | Chemical structural formulae | Inhibition constant (Ki)/ half maximal inhibitory concentration (IC50) | Dissociation constant (Kd) | Concentration of binding components (Bmax) | Binding potential (BP=Bmax/Kd) | On-target | Off-target |
| --- | --- | --- | --- | --- | --- | --- | --- |
| [11C]-PBB3(Maruyama et al., 2013;Ono et al., 2017) | 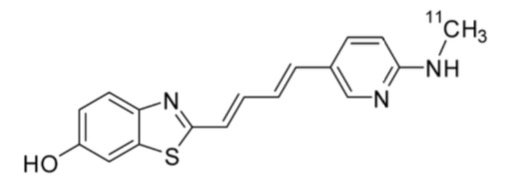 | AD: 1.3 nM, 23.5 nM;  PSP: 5.9 nM | AD: 6.3 nM  PSP: 4.8 nM | AD: 4303pmol/g  PSP: 589.7pmol/g | AD: 687.4  PSP: 122.5 | Binding to NFT, neuritic plaques, primitive plaques, Pick bodies, tau inclusions in PiD, PSP and CBD | Binding to dense core amyloid plaques and diffuse amyloid-beta deposits, astrocytic plaques |
| [^18^F]-AV-1451(Marquie et al., 2015;Hostetler et al., 2016;Ono et al., 2017;Tsai et al., 2019) | 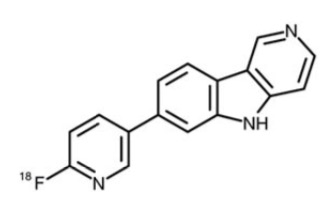 | AD: 97.2 nM,0.3 nM;  PSP: 3.3 nM | AD: 0.63-3.72 nM/14.6 nM  Non-AD: 0.79-10.3 nM | AD: 15-119.7nM;  Non-AD: 61-190 nM | AD: 11-109;  Non-AD: 5.9-40 | Binding to PHF-tau, pretangles, mature tangles, neuritic and primitive plaques (to a limited extent) | Binding to MAO-A, MAO-B, dense core amyloid plaques, melanin- and neuromelanin-containing cells and some weaker binding to blood component |
| [^18^F]-THK5117(Lemoine et al., 2017) | 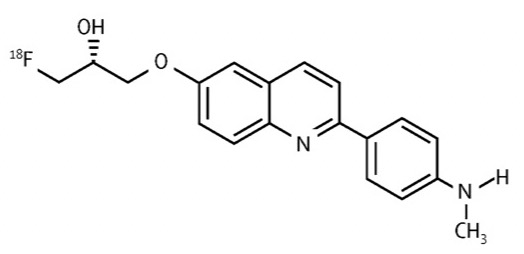 | AD: 0.001 nM; 0.0005 nM;10.5–27.4 nM; 750-800 nM | AD: 2.2–3.1; 23.6–34.6;5.19– 11.5;5.2 | AD: 250; 1226–1416; 338 |  | Binding to pretangles, PHF-tau, NFT, neuritic plaques, argyrophillic grains, argyrophilic threads, globose tangles | Binding to MAO-B |
| [^18^F]-THK5351(Lemoine et al., 2017;Ono et al., 2017) | 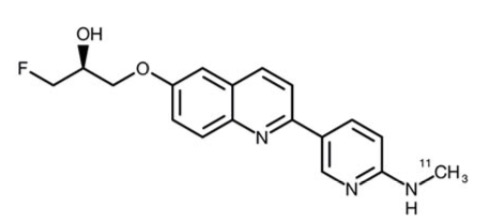 | AD: 0.1 pM; 16 nM | AD: 5.6 nM; 1 nM | AD: 76 pmol/g; 40 pmol/g | AD: 13.6;  40 | Binding to NFT, thread like structures in white matter, tufted astrocytes | Binding to MAO-B |
| Second-generation tau PET tracer |  |  |  |  |  |  |  |
| [^18^F]- APN-1607 ([^18^F]-PM-PBB3)(Tagai et al., 2020) | 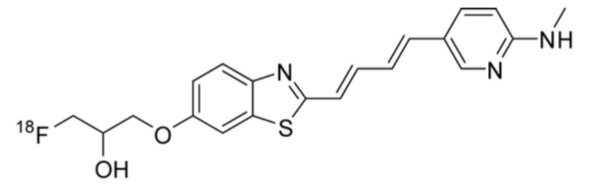 | 379.1 nM | AD: 7.63 nM; PSP: 3.44 nM | AD: 5743 pmol/g; PSP: 688.2 pmol/g | AD: 752.7; PSP: 199.9 | Binding to NFT, neuropil threads, and dystrophic neurites encompassing neuritic plaques in AD; 3R-tau in PiD; 4R-tau in PSP, CBD | Barely off-target binding to MAO-A and MAO-B |
| [^18^F]-MK6240(Hostetler et al., 2016;Aguero et al., 2019) | 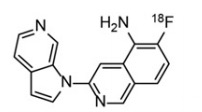 | AD: 0.36 nM | AD: 0.14-0.38nM | AD: 7.8-93.4nM | AD: 56-246 | Binding to NFT in AD | Binding to neuromelanin and melanin-containing cells, and some weaker binding to areas of hemorrhage.  Barely off-target binding to MAO-B and not to MAO-A. |
| [^18^F]-RO948(Honer et al., 2018;Smith et al., 2020) | 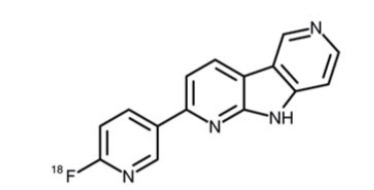 | IC_50_: 18.5nM |  |  |  | Binding to NFT, neuropil threads  in AD | No off-target bindings to Aβ. Off-target bindings to skull and menings. |
| [^18^F]-PI2620(Kroth et al., 2019) | 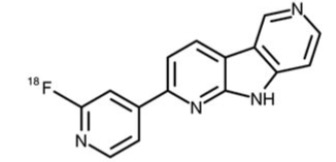 | IC_50_: 1.8nM |  |  |  | Binding to NFT in AD; 4R-tau in PSP; 3R-tau in PiD | No off-target bindings to MAO-A, MAO-B, Aβ.  Off-target bindings in the pars compacta of human sections from the substantia nigra |

AD= Alzheimer’s disease; PSP= Progressive supranuclear palsy; PiD= Pick’s disease; CBD= Corticobasal degeneration; NFT= Neurofibrillary tangles; PHF-tau= Paired helical filament-tau; 3R-tau= Three microtubule-binding domains-tau; 4R-tau=Four microtubule-binding domains; MAO= Monoamine oxidase

References:

Aguero, C., Dhaynaut, M., Normandin, M.D., Amaral, A.C., Guehl, N.J., Neelamegam, R., Marquie, M., Johnson, K.A., El Fakhri, G., Frosch, M.P., and Gomez-Isla, T. (2019). Autoradiography validation of novel tau PET tracer [F-18]-MK-6240 on human postmortem brain tissue. *Acta Neuropathol Commun* 7**,** 37.

Honer, M., Gobbi, L., Knust, H., Kuwabara, H., Muri, D., Koerner, M., Valentine, H., Dannals, R.F., Wong, D.F., and Borroni, E. (2018). Preclinical Evaluation of (18)F-RO6958948, (11)C-RO6931643, and (11)C-RO6924963 as Novel PET Radiotracers for Imaging Tau Aggregates in Alzheimer Disease. *J Nucl Med* 59**,** 675-681.

Hostetler, E.D., Walji, A.M., Zeng, Z., Miller, P., Bennacef, I., Salinas, C., Connolly, B., Gantert, L., Haley, H., Holahan, M., Purcell, M., Riffel, K., Lohith, T.G., Coleman, P., Soriano, A., Ogawa, A., Xu, S., Zhang, X., Joshi, E., Della Rocca, J., Hesk, D., Schenk, D.J., and Evelhoch, J.L. (2016). Preclinical Characterization of 18F-MK-6240, a Promising PET Tracer for In Vivo Quantification of Human Neurofibrillary Tangles. *J Nucl Med* 57**,** 1599-1606.

Kroth, H., Oden, F., Molette, J., Schieferstein, H., Capotosti, F., Mueller, A., Berndt, M., Schmitt-Willich, H., Darmency, V., Gabellieri, E., Boudou, C., Juergens, T., Varisco, Y., Vokali, E., Hickman, D.T., Tamagnan, G., Pfeifer, A., Dinkelborg, L., Muhs, A., and Stephens, A. (2019). Discovery and preclinical characterization of [(18)F]PI-2620, a next-generation tau PET tracer for the assessment of tau pathology in Alzheimer's disease and other tauopathies. *Eur J Nucl Med Mol Imaging* 46**,** 2178-2189.

Lemoine, L., Gillberg, P.G., Svedberg, M., Stepanov, V., Jia, Z., Huang, J., Nag, S., Tian, H., Ghetti, B., Okamura, N., Higuchi, M., Halldin, C., and Nordberg, A. (2017). Comparative binding properties of the tau PET tracers THK5117, THK5351, PBB3, and T807 in postmortem Alzheimer brains. *Alzheimers Res Ther* 9**,** 96.

Marquie, M., Normandin, M.D., Vanderburg, C.R., Costantino, I.M., Bien, E.A., Rycyna, L.G., Klunk, W.E., Mathis, C.A., Ikonomovic, M.D., Debnath, M.L., Vasdev, N., Dickerson, B.C., Gomperts, S.N., Growdon, J.H., Johnson, K.A., Frosch, M.P., Hyman, B.T., and Gomez-Isla, T. (2015). Validating novel tau positron emission tomography tracer [F-18]-AV-1451 (T807) on postmortem brain tissue. *Ann Neurol* 78**,** 787-800.

Maruyama, M., Shimada, H., Suhara, T., Shinotoh, H., Ji, B., Maeda, J., Zhang, M.R., Trojanowski, J.Q., Lee, V.M., Ono, M., Masamoto, K., Takano, H., Sahara, N., Iwata, N., Okamura, N., Furumoto, S., Kudo, Y., Chang, Q., Saido, T.C., Takashima, A., Lewis, J., Jang, M.K., Aoki, I., Ito, H., and Higuchi, M. (2013). Imaging of tau pathology in a tauopathy mouse model and in Alzheimer patients compared to normal controls. *Neuron* 79**,** 1094-1108.

Ono, M., Sahara, N., Kumata, K., Ji, B., Ni, R., Koga, S., Dickson, D.W., Trojanowski, J.Q., Lee, V.M., Yoshida, M., Hozumi, I., Yoshiyama, Y., Van Swieten, J.C., Nordberg, A., Suhara, T., Zhang, M.R., and Higuchi, M. (2017). Distinct binding of PET ligands PBB3 and AV-1451 to tau fibril strains in neurodegenerative tauopathies. *Brain* 140**,** 764-780.

Smith, R., Schöll, M., Leuzy, A., Jögi, J., Ohlsson, T., Strandberg, O., and Hansson, O. (2020). Head-to-head comparison of tau positron emission tomography tracers [(18)F]flortaucipir and [(18)F]RO948. *Eur J Nucl Med Mol Imaging* 47**,** 342-354.

Tagai, K., Ono, M., Kubota, M., Kitamura, S., Takahata, K., Seki, C., Takado, Y., Shinotoh, H., Sano, Y., Matsuoka, K., Takuwa, H., Shimojo, M., Takahashi, M., Kawamura, K., Kikuchi, T., Okada, M., Akiyama, H., Suzuki, H., Onaya, M., Takeda, T., Arai, K., Arai, N., Araki, N., Saito, Y., Kimura, Y., Ichise, M., Tomita, Y., Zhang, M.-R., Suhara, T., Shigeta, M., Sahara, N., Higuchi, M., and Shimada, H. (2020). High-contrast in-vivo imaging of tau pathologies in Alzheimer's and non-Alzheimer's disease tauopathies. *medRxiv***,** 2020.2003.2005.20028407.

Tsai, R.M., Bejanin, A., Lesman-Segev, O., Lajoie, R., Visani, A., Bourakova, V., O'neil, J.P., Janabi, M., Baker, S., Lee, S.E., Perry, D.C., Bajorek, L., Karydas, A., Spina, S., Grinberg, L.T., Seeley, W.W., Ramos, E.M., Coppola, G., Gorno-Tempini, M.L., Miller, B.L., Rosen, H.J., Jagust, W., Boxer, A.L., and Rabinovici, G.D. (2019). (18)F-flortaucipir (AV-1451) tau PET in frontotemporal dementia syndromes. *Alzheimers Res Ther* 11**,** 13.
